# Effects of carbon dioxide on physical and cognitive performance in a simulated spacesuit contingency scenario

**DOI:** 10.1038/s41526-026-00589-x

**Published:** 2026-04-21

**Authors:** N. Keller, S. Thoolen, B. Siders, P. Estep, R. Scully, B. D. Levine, T. Babb, J. Pawelczyk, A. Baughman, M. Basner, M. Young, L. Chappell, J. Norcross, K. Marshall-Goebel, A. Garbino

**Affiliations:** 1 KBR, Inc, Houston, TX USA; 2 GeoControl Systems, Houston, TX USA; 3 Aegis Aerospace Inc, Webster, TX USA; 4 University of Texas Southwestern Medical Center, Dallas, TX USA; 5 Institute for Exercise and Environmental Medicine, Dallas, TX USA; 6 Penn State University, University Park, Philadelphia, PA USA; 7 University of Pennsylvania, Philadelphia, PA USA; 8 NASA Johnson Space Center, Houston, TX USA

**Keywords:** Medical research, Neuroscience

## Abstract

Crewmembers on future Lunar and Mars surface missions will perform numerous surface exploration extravehicular activities (EVAs). NASA standards dictate a nominal upper limit to the inspired partial pressure of CO_2_ (P_I_CO_2_) of 15 mmHg in spacesuits; however, there is no suited P_I_CO_2_ standard for emergency or contingency EVA scenarios. A laboratory-based study was conducted to characterize physical and cognitive performance, and self-reported symptomology during a simulated emergency one-hour EVA return to habitat scenario while being exposed to different P_I_CO_2_ levels. Fifteen healthy subjects (12 M/3 F) underwent 7 testing conditions (P_I_CO_2_ of 0, 5, 10, 15, 20, 25, and 30 mmHg) using a single-blinded, repeated-measures, counterbalanced design. All subjects completed the simulated contingency EVA scenario at all P_I_CO_2_ levels. Although statistically detectable dose-responses were observed in several variables, these changes did not reach levels associated with clinically or operationally relevant impairment during the one-hour exposures up to 30 mmHg P_I_CO_2_.

## Introduction

During human exploration missions to the Moon and Mars, crewmembers will undergo frequent extravehicular activities (EVAs)—or spacewalks—on the planetary surface to conduct science, deploy payloads, and explore. Spacesuits worn during EVAs function as enclosed life-support systems that must maintain a stable environment while the crewmember produces heat, humidity, and metabolic waste gas such as carbon dioxide (CO_2_). The accumulation of CO_2_ in spacesuits and habitats is a concern due to its potential negative impact on performance and health^1–3^.

The ability of the spacesuit to adequately control CO_2_ levels during an EVA depends on a variety of factors, including the geometry of the suit volume, configuration of ventilation loops, flow rates of the breathing gas, efficacy of the CO_2_ removal system, and the occupant’s rate of metabolic CO_2_ production (V̇CO_2_). In certain off-nominal “contingency” EVA scenarios, such as failure of the suit’s CO_2_ removal system, a purge valve must be opened in the suit helmet to exhaust the suit’s atmosphere to vacuum and prevent CO_2_ accumulation. Based on the suit’s design, the size of the oxygen (O_2_) gas supply, and flow rate through the purge valve, these contingency scenarios would afford crewmembers approximately one hour to return safely to their habitat before exhausting available suit consumables. Lunar surface EVA operations are planned such that a crewmember can traverse back to their habitat at 2 km/h with an assumed average metabolic rate of less than 1200 BTU/h (303 kcal/h). The transition of the suit ventilation into an open loop architecture, however, changes the efficacy of CO_2_ removal from the suit due to altered gas flow dynamics^4^. CO_2_ levels will also begin to rise faster if metabolic rate exceeds physical workload limits. Therefore, there is a risk in these contingency scenarios that CO_2_ may accumulate to levels that could compromise a crewmember’s ability to safely return to their habitat.

For nominal operations, NASA’s health standards for human spaceflight (NASA-STD-3001, Volume 2)^5^ currently requires spacesuits to limit the inspired partial pressure of CO_2_ (P_I_CO_2_) to a maximum of 15 mmHg, permitting exposure time limits at lower CO_2_ levels, ranging from 30 min at a P_I_CO_2_ between 12.5 and 15 mmHg, to 7 hours at a P_I_CO_2_ between 4 and 7 mmHg^5,6^. As P_I_CO_2_ increases, it interferes with the body’s ability to maintain stable arterial CO_2_ tension in the blood and other tissues, which is essential for homeostasis and proper cellular function^6^. In an attempt to limit increases in arterial CO_2_ (P_a_CO_2_), an acute chemosensory response typically increases ventilation to facilitate diffusion of CO_2_ into the alveoli, and removal from the body via the airways. The inhalation of extraneous CO_2_ opposes the efficacy of this respiratory response, and may eventually lead to arterial hypercapnia and acute respiratory acidosis at high enough CO_2_ exposures^6^. Depending on dose and duration, symptoms from hypercapnia could include shortness of breath, headaches, dizziness, anxiety, and confusion to unconsciousness, seizures, and death^2,7–10^.

While some terrestrial functional performance data exists for acute P_I_CO_2_ exposures greater than 15 mmHg^11–20^, there is uncertainty and inconsistency on the performance impacts of these elevated P_I_CO_2_ levels, especially in association with physical activity. Some studies^11–13,18^ suggest that P_I_CO_2_ levels of 20 mmHg (some as high as 28.5 mmHg^16,19^) are tolerable for an hour without major physical and cognitive performance impacts, while other data^8,14,20–22^ imply that such levels could unacceptably impact contingency EVA scenarios. Impairments in cognitive tasks that may be critical in EVA operations, such as attention and complex decision-making, have been detected at acute P_I_CO_2_ exposures as low as 1.1–1.8 mmHg^8,21–23^, while others did not find cognitive impairments in reasoning, short-term memory, or psychomotor performance at P_I_CO_2_ levels as high as 32–38 mmHg^15,17^. Notably, there can be substantial individual variability in response to elevated P_I_CO_2_, and possible synergistic effects of the spaceflight environment with elevated P_I_CO_2_ are unknown. in both physical and cognitive domains. As such, the maximum level of CO_2_ exposure at which crewmembers could safely return to the habitat in an EVA contingency scenario remains undetermined.

Here, a laboratory-based study was conducted to generate dose-response relationships that characterize physical and cognitive performance as well as self-reported symptomology when subjected to 7 P_I_CO_2_ levels up to 30 mmHg during a virtual reality (VR)-simulated emergency EVA contingency scenario requiring crew to return to their habitat in one hour. It was hypothesized that performance would be reduced with increasing CO_2_ exposure, to a point that would be operationally unacceptable for contingency EVA situations.

## Results

In total, 15 healthy subjects (3 female) participated in the study (mean ± standard deviation; age: 34.6 ± 5.6 years, weight: 77.33 ± 7.82 kg, BMI: 23.99 ± 2.08 kg/m², maximum O_2_ uptake [VO_2peak_]: 42.7 ± 8.5 ml/min/kg, and first ventilatory threshold (VT1): 50.7 ± 11.3 L/min). All subjects were able to complete the 1-h, 2 km contingency EVA “walk back” simulation at all 7 levels of P_I_CO_2_. Table 1 summarizes grouped physical and cognitive responses at each level of P_I_CO_2_ and main effects of the intervention along with direction of any detected trend.

**Table 1:** Main Effects and group mean responses ± 1 SE by P_I_CO_2_ level during simulated EVA walk back

	P_I_CO_2_ Level (mmHg)							Main Effects	
	0	5	10	15	20	25	30	*p*	Trend
*Traverse*									
Normalized Walking Speed (%)	71.0 ± 3.4	69.7 ± 3.7	68.5 ± 3.9	69.0 ± 5.3	71.6 ± 4.2	68.7 ± 4.1	69.8 ± 4.1	<0.001	↓
Total Distance (m)	2872 ± 109.2	2863.1 ± 90.2	2886.6 ± 110.2	2886.8 ± 125.2	2906.6 ± 110.9	2851.6 ± 100.5	2822.2 ± 117.1	0.73	None
*Physiological*									
Oxygen Consumption (mL/min)	1054.2 ± 25.5	1026.1 ± 39.7	1060.3 ± 25.4	1058.6 ± 42.3	1022.1 ± 22.0	1034 ± 21.0	1012.9 ± 25.7	<0.001	↓
Minute Ventilation (L/min)	28.64 ± 0.77	31.61 ± 0.94*	35.52 ± 1.01*	38.27 ± 1.66*	42.33 ± 1.19*	46.37 ± 1.70*	52.67 ± 1.79*	<0.001	↑
Tidal Volume (L)	1.33 ± 0.03	1.47 ± 0.05*	1.55 ± 0.04*	1.68 ± 0.06*	1.82 ± 0.05*	2.04 ± 0.07*	2.19 ± 0.06*	<0.001	↑
Respiratory Rate (bpm)	22.23 ± 0.67	22.54 ± 0.68	23.80 ± 0.78	23.87 ± 0.75*	24.13 ± 0.85*	23.72 ± 0.86*	25.16 ± 0.96*	<0.001	↑
End-Tidal CO_2_ (mmHg)	39.8 ± 0.8	40.5 ± 0.9*	42.1 ± 0.8*	42.7 ± 0.8*	46.1 ± 0.9*	48.4 ± 0.8*	49.7 ± 0.8*	0.001	↑
Transcutaneous CO_2_ (mmHg)	38.1 ± 0.9	39.0 ± 0.8	39.8 ± 0.8	40.1 ± 0.6*	42.2 ± 0.8*	44.1 ± 0.6*	44.9 ± 0.6*	<0.001	↑
Oxygen Saturation (%)	97.9 ± 0.1	98.3 ± 0.2	98.3 ± 0.1*	98.5 ± 0.2*	98.3 ± 0.2*	98.4 ± 0.2*	98.7 ± 0.1*	0.03	↑
Heart Rate (bpm)	93.4 ± 3.7	96.2 ± 2.8	95 ± 3.4*	95.2 ± 3*	97.5 ± 3.5*	101.3 ± 3.7*	103 ± 3.4*	<0.001	↑
*Cognitive*									
DSST Accuracy (%)	97.49 ± 0.89	97.47 ± 0.61	98.1 ± 0.52	98.03 ± 0.62	97.18 ± 0.93	97.89 ± 0.61	97.72 ± 0.62	0.02	↑
DSST Reaction Time (sec)	1.07 ± 0.04	1.12 ± 0.04	1.09 ± 0.05	1.11 ± 0.05	1.17 ± 0.05	1.15 ± 0.05	1.14 ± 0.05	<0.001	↑
DSST Throughput (Correct/min)	54.96 ± 1.56	52.88 ± 1.4	54.68 ± 1.27	53.77 ± 1.08	53.14 ± 1.38	52.03 ± 1.1	52.09 ± 1.04	<0.001	↓
Alert Light Functional Task time (sec)	0.79 ± 0.05	0.78 ± 0.05	0.73 ± 0.04	0.82 ± 0.07	0.79 ± 0.06	0.82 ± 0.05	0.74 ± 0.06	0.88	None

Mixed model main effects significance for P_I_CO_2_ dose-response effects are shown on the right, and the direction of any corresponding trend. * indicate significant differences (*p* < 0.05) in post-hoc contrasts from the same-day’s walking baseline mean value. Data are shown as measured group mean ± standard error. Note: Alert Light Functional Task time means were log-transformed to meet normality assumptions of the mixed model.

*P*_*I*_*CO*_2_ inspired partial pressure of CO2, *bpm* beats per minute, *DSST* Digit-Symbol Substitution Test.

### Physiological responses

Physical workload, as measured via oxygen consumption rate (V̇O_2_), averaged 1005 ± 30 mL/min across all tests. Subjects were prescribed a maximal VO_2_ of 1300 mL/min, but most subjects demonstrated a preference to work below this limit. Oxygen consumption tended to decrease with increasing P_I_CO_2_ (main effect *p* < 0.001); however, as seen on Table 1, no significant differences were detected between the CO_2_ exposure phase and the walking baseline at ambient air measured on the same day, for any P_I_CO_2_ level. Similarly, Walking Speed demonstrated a marginal yet statistically significant decrease among subjects with increasing P_I_CO_2_ as a normalized percentage of their prescribed speed range (*p* < 0.001); however, as with V̇O_2_, post-hoc contrasts showed no differences at any level when compared to same-day walking baseline levels and the total distance traversed remained unchanged across P_I_CO_2_ levels (*p* = 0.73, Table 1).

**Fig. 1:**
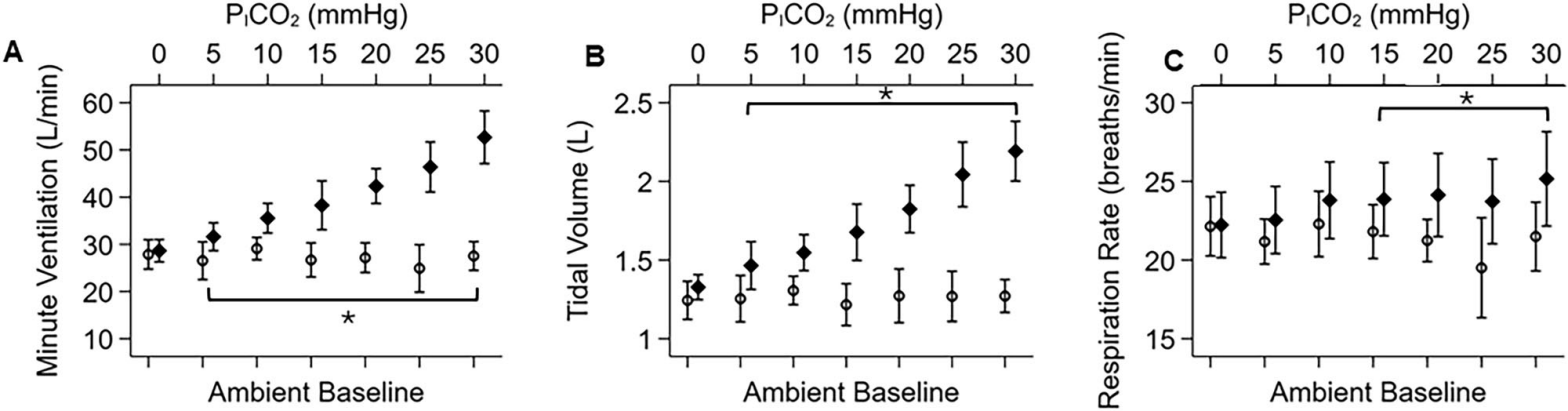
Mean ventilatory responses across all P_I_CO_2_ levels (diamonds) and walking baseline measures (ambient air, circles). Minute ventilation (**A**), total volume (**B**), and respiration rate (**C**). Asterisks (*) indicate significant differences detected (*p* < 0.05) during post hoc contrasts between means measured during the 1-h intervention period and the walking baseline at ambient air. Data are shown as mean ± 95% confidence intervals.

Minute ventilation increased significantly with P_I_CO_2_ (*p* < 0.001, +25.1 L/min (CI: 20.8–29.5)) as compared with the maximal exposure of 30 mmHg, as will be reported consistently throughout this text. This change was primarily driven by concomitant increases in tidal volume (*p* < 0.001, +0.9 L (CI: 0.8–1.1)), and less robust increases in respiratory rate (*p* < 0.001, +3.7 breaths/min (CI: 1.9–5.5)). For context that will be discussed in the following section, the mean first ventilatory threshold (VT1) determined during baseline VO_2peak_ testing was 50.7 ± 11.3 L/min, and mean minute ventilation at the highest level of CO_2_ (30 mmHg) was 52.8 ± 1.8 L/min. VCO_2_ demonstrated no statistically significant change across CO_2_ levels (*p* = 0.32). Ventilatory responses across all P_I_CO_2_ levels are shown in Fig. 1.

The partial pressure of end-tidal CO_2_ (PetCO_2_) increased significantly (*p* < 0.001, +10.9 mmHg (CI: 9.0–12.9)) along with transcutaneous CO_2_ (TcCO_2_) (*p* < 0.001, +5.7 mmHg (CI: 4.3–7.1)) with CO_2_ exposure. Oxygen saturation increased as a function of P_I_CO_2_ level (*p* = 0.03, +0.8% (CI: 0.5–1.0)). Heart rate also demonstrated a statistically significant increase as a function of P_I_CO_2_ (*p* < 0.001, +10.5 beats/min (CI: 6.3–14.6)).

### Cognitive performance

**Fig. 2:**
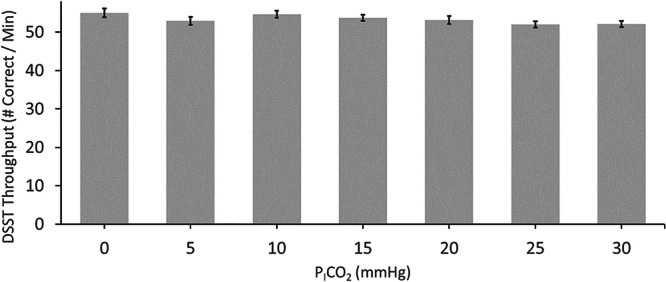
Digit symbol substitution test (DSST) throughput across P_I_CO_2_ levels during the 1-h simulated EVA walk back. The a priori, operationally relevant threshold was 49.5 (number of correct responses per minute, 10% decline from baseline). While DSST throughput declined as a function of P_I_CO_2_ (*p* < 0.001), it did not reach the level of operational relevance. Data are shown as group means ± CI.

The percentage of correct responses on the Digit Symbol Substitution Test (DSST) increased with P_I_CO_2_ (*p* = 0.02, +0.4% (CI: 0.1–0.7)). DSST reaction time similarly demonstrated a significant increase (*p* < 0.001, +0.06 s (CI: 0.01–0.9)). DSST throughput, a measure inclusive of both accuracy and response time, as measured by number of correct responses per minute, decreased significantly with P_I_CO_2_ levels (*p* < 0.001, –3.0 correct/min (CI: 1.2–4.8)), starting at 55.0 ± 1.6 correct/min, and falling to 52.1 ± 1.0 correct/min, a decline of 5.2% (Fig. 2). The a priori threshold for an operationally relevant decrement in DSST throughput performance was set at 10%.

Alert Light Functional Task times did not show a significant effect of P_I_CO_2_ levels (*p* = 0.88, Table 1).

### Symptoms and self-assessed performance

**Fig. 3:**
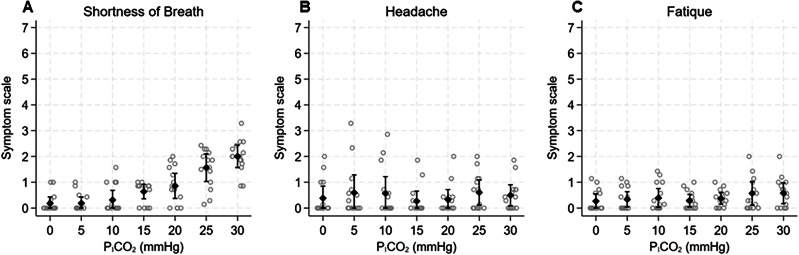
Symptoms demonstrating significant dose-response effects of P_I_CO_2_. Shortness of breath (**A**), headache (**B**), and fatigue (**C**). Data are shown as group means ± 95% confidence intervals (black closed diamonds), as well as individual subject means (gray open circles) for each P_I_CO_2_ level. A Symptom scale score of 0 indicates “No Symptoms” and 7 indicates “Intolerable Symptoms”.

Shortness of breath (*p* < 0.001), headache (*p* < 0.001), and fatigue (*p* < 0.001) symptoms demonstrated increasing severity with P_I_CO_2_, while nausea, dizziness, numbness, tingling, and euphoria did not demonstrate significant changes. Symptoms with detectable changes are show in Fig. 3. The maximum observed shortness of breath score was 4 on the Likert scale from 0 (“no symptoms”) to 7 (“intolerable”), which was seen in 3% of all reports at a P_I_CO_2_ of 30 mmHg. The maximum observed fatigue score was 3, which was seen in 3.3% of all reports at a P_I_CO_2_ of 25 mmHg and in 3.1% of all reports at 30 mmHg.

**Fig. 4:**
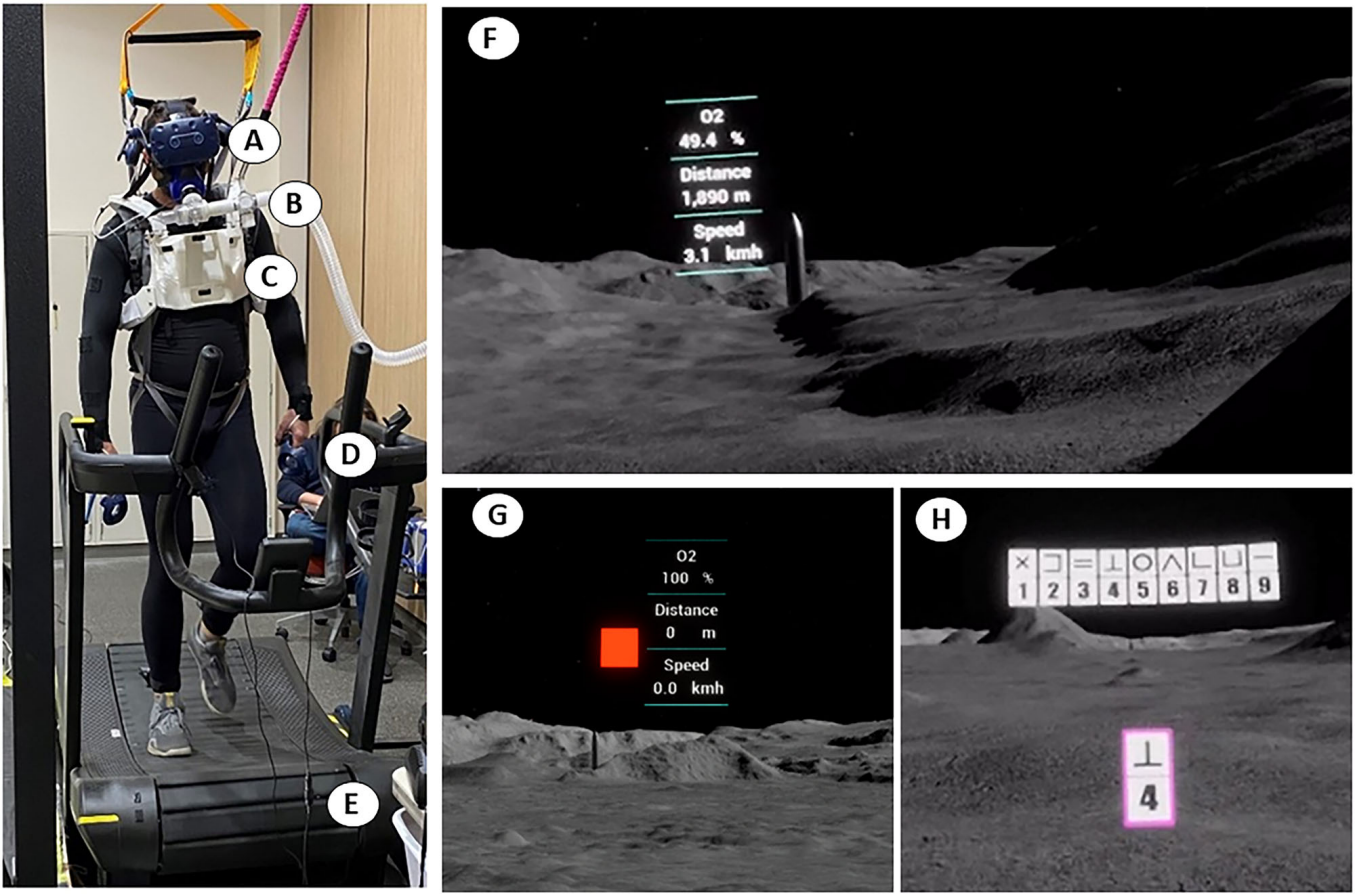
EVA simulation equipment setup. Left panel: **A** Vive Pro Eye virtual reality headset, **B** breathing gas intake hose, **C** sized 3D-printed simulated spacesuit hard upper torso, (**D**) VR hand controllers for completing VR-driven cognitive tests, **E** self-propelled passive treadmill. Panel **F**: nominal VR view and heads-up display. Panel **G**: red light shown from Alert Light Functional Task. Panel **H**: Digit Symbol Substitution Test display (replacing the nominal heads-up display).

The headache symptom analysis was confounded by persistent reports of subject discomfort with the testing hardware around the head, seen in Fig. 4A. During the daily debrief, subjects attributed their headache symptom to the VR headset, Drager breathing mask, or the interaction of the two, in most reports of headache incidence. Mid-study anecdotal findings of post-testing headaches prompted an ad hoc surveillance of active and completed subjects. Five out of 15 subjects (33%) reported at least one episode of post-testing headache, with a severity ranging from 0.5 to 6 on the 0–7 Likert scale. Four of the subjects were able to determine that the symptom occurred after either their 25 or 30 mmHg exposure, while in the remaining case the subject was not able to confidently recall the test date when their headache occurred.

Self-assessed ratings of performance were measured on a scale from 1 (“incapable”) to 7 (“optimal”) immediately following each symptom survey. Self-assessed speed performance ratings demonstrated no trends across P_I_CO_2_ levels. Self-assessed endurance performance significantly decreased with P_I_CO_2_ (*p* = 0.03), with 5 reports of “6” at the 0 mmHg control level, and 23 reports of “6” at the 30 mmHg highest level, and 3 or fewer total reports of “5” or “4” at each non-0 level. Self-assessed cognitive performance ratings decreased (*p* < 0.01), worsening from 5 reports of “5” (and no worse) at the 0 mmHg condition, up to 30 reports of “5” and 1 report of “4” at the 30 mmHg condition.

## Discussion

The current work assesses the physiological, cognitive, symptom, and performance impacts of increasing levels of inhaled carbon dioxide during a simulated emergency EVA “walk back” scenario lasting 1 h. Specifically, this scenario explores elevated CO_2_ levels in a catastrophic failure of the suit CO_2_ removal capability, where survival depends on an immediate return to the habitat. As implemented in the laboratory for this test, the scenario required subjects to ambulate on a passive treadmill within an individually prescribed range of speeds while intermittently responding to visual and verbal cognitive, symptom, and performance-related prompts. All 15 subjects were able to complete the 1-h simulated EVA walk back to their habitat with minimal cognitive and physical impacts at levels of P_I_CO_2_ up to 30 mmHg. Although statistically detectable dose-responses were observed across several key variables, these changes did not reach levels associated with clinically or operationally relevant impairment during a contingency scenario. The present findings are relevant to the development of EVA operational guidelines as well as spacesuit hardware requirements and design.

Physiological responses to hypercapnia were consistent with a low to moderate physical workload in a normal setting. Across the range of 0–30 mmHg P_I_CO_2_, the mean partial pressure of end-tidal CO_2_ increased by 10.9 mmHg, while the mean transcutaneous CO_2_ across the same range only increased by 5.7 mmHg. Compared to the amount of inspired CO_2_, these rises are consistent with literature at the levels tested and likely reflect ventilatory compensation and buffering capacity of CO_2_ by bicarbonate in the blood^24–26^. While heart rate demonstrated a dose-response effect of hypercapnia, the effect was mild, increasing less than 10 beats per minute on average between the control condition and the highest P_I_CO_2_ level. Although the ease of measuring heart rate makes it a common metric for crew health assessment in spacesuits, the magnitudes of heart rate increases seen in this study demonstrate that it does not have any meaningful predictive value for an elevated CO_2_ exposure scenario, especially given the many other factors that affect heart rate. Tidal volume was the most significant and consistent respiratory response. At its maximum response, minute ventilation reached a mean of 52.6 ± 1.8 L/min. For comparison, the average minute ventilation at the first ventilatory threshold (VT1; typically shown as oxygen consumption, but shown as minute ventilation here for illustrative purposes) of all subjects was found to be 50.7 ± 11.3 L/min. Healthy individuals can typically maintain a VT1 equivalent of physical workload for several hours^27,28^, and the current subjects generally reached this level with ambulation coupled with a P_I_CO_2_ of 30 mmHg. Further, no decreases in oxygen saturation were observed, despite indications of increased arterial CO_2_ as measured through PetCO_2_ and TcCO_2_. Here, further evidence is provided that increasing CO_2_ tension in the blood does not result in an overall decrease of oxygenation at the levels tested as the ventilatory response is sufficient to prevent this^24^.

As seen in Table 1, Subjects self-selected a pace close to 70% of their prescribed maximum walking speed, though this maximum prescription varied between 2.6 and 4.6 km/h depending on the individual. This variability in the range of allowable speeds confounds inferences of effects of CO_2_ exposure on total distanced traversed. Still, while such inferences appear to be inconsequential insofar as the 2 km contingency scenario is concerned, the potential for CO_2_ exposure to impede a physical traverse may impact longer traverses or those at higher physical workloads with a greater production of endogenous CO_2_, and thereby increase the likelihood of hypercapnic symptoms at lower levels of P_I_CO_2_ than seen here^13^. It may also be speculated that while the physiological response seen here seemed adequately protective to accomplishing the task, a longer exposure time and/or a greater volume of CO_2_ exposure may tax these systems beyond their capabilities, although the possibility of adaption should also be considered^9,11,16^.

Cognitive effects of hypercapnia were minimal in the 1 h simulated contingency EVA. Among the outputs of the DSST_VR_ cognitive test, “Throughput” is the primary performance metric as it incorporates both accuracy and speed domains^29,30^. Prior to testing, an arbitrary but conservative a priori threshold of a 10% decrease in DSST Throughput relative to baseline was determined to be an operationally relevant decrement, and this was not reached, though mild (3–5%) decreases in performance were observed across the range of P_I_CO_2_ conditions. As no effects were detected in response time of the Alert Light Functional Task either, we reject our hypothesis that cognitive performance would be reduced to operationally relevant levels as a function of P_I_CO_2_ up to 30 mmHg at the physical workload tested. These findings parallel other work on acute CO_2_ exposures^31–36^, although findings may differ with respect to longer duration or chronic exposures.

Self-assessed ratings of hypercapnia symptoms and ratings of performance of the contingency task indicated that the protocol was well tolerated. The dose-response relationship of Shortness of Breath to P_I_CO_2_ contextualizes the current finding of Tidal Volume as the most robust physiological response and supports previous literature demonstrating dyspnea and other synonymous symptoms as the most common symptom of elevated CO_2_ exposure^37^. The findings of mild headache symptoms associated with increasing P_I_CO_2_ are known, however, the headache data herein is confounded by consistent reports of subject discomfort with the hardware around the head (VR headset, breathing mask) used during the simulated EVA scenario. While headaches are a clear symptom that subjects may or may not report, dyspnea is a clear symptom an observer could detect. The finding of a mild increase in fatigue and mild decrease in self-reported physical endurance may be attributable to the effort of increased respiration, especially given that VO_2_, an indicator of physical workload, decreased marginally rather than increase. Similarly, the mild decrease in self-reported cognitive performance is belied by the lack of effects seen in actual cognitive performance, although this could be explained by a subjective sensation of increased cognitive demand that is nonetheless compensated by the subjects’ cognitive reserve^38^. Although anxiety and hypercapnia have been associated previously, doses employed in the current work are not believed to have elevated anxiety to such a degree that would impact performance, though admittedly anxiety was not measured^39,40^. Besides headache, the finding of no trend among other symptoms nor self-reported speed performance at the P_I_CO_2_ levels tested indicate that such levels are well tolerated by subjects.

While the current study employed a highly operationally-relevant environment for testing the ability of subjects to complete a 2 km contingency EVA “walk back” scenario, there are three key environmental factors which could not be reproduced and thereby limit the operational applicability: (1) EVA’s are conducted within the spacesuit environment using a nominal 4.3 psia of atmospheric pressure at near-100% O_2_ (mildly hyperoxic); (2) EVA’s on the Moon and Mars will be conducted at 1/6th and 3/8ths of Earth’s gravity, respectively, and (3) Subjects were not physiologically deconditioned to a degree seen in spaceflight missions. These spaceflight-specific conditions, considered together or separately, may influence the nominal response to hypercapnia to an unknown degree. For this reason, partial pressures of CO_2_ were used to ascertain effects of precise dosages rather than concentrations, and metabolic rates were based on Apollo EVA workloads, prototype space suit development and limited to expected EVA suit thermal (heat rejection) limitations. These controls permit the translation of the current data to Lunar and Martian EVA environments. It is theoretically possible that (perhaps as a result of deconditioning) the minimum ambulation speed necessary to reach safe habitat from a 2 km starting distance would result in a metabolic workload beyond the capability of the suit, but such a scenario is unlikely and currently mitigated with exercise countermeasures. A mildly hypobaric, hyperoxic environment (3.7 psia O_2_ when the space suit is utilizing the purge valve in the tested scenario), also coupled with potential deconditioning, may also bias the effects seen here but this would need to be investigated in future work. Also, the attentional resources required by the contingency tasks were reported by subjects to be very minimal. It could not be determined if this had a detrimental effect on test performance, but a more compelling environment, free movement via a multi-dimensional treadmill, or the inclusion of more EVA-relevant tasks, may demonstrate different outcomes.

Additionally, hardware fit on the head could have impacted headache symptomology and affected results that may not be directly attributable to CO_2_ exposure. While findings demonstrated a mild increasing dose-response trend of P_I_CO_2_, a separate analysis of the effect of experiment order (#1–7) on the headache symptom showed a much stronger *decreasing* dose-response trend, suggesting that subjects acclimated to the testing environment in a way that confounds attribution of the headache symptom to CO_2_ exposure alone. Subjects were informed of the possibility of VR-associated symptoms yet selected to join the study, and were given a complete familiarization session to learn and fit the head-mounted hardware to their comfort level, yet it is still possible that other, unmeasured, unreported, symptoms associated with VR exposure such as simulator sickness, eye strain (both of which could lead to headache symptoms), or competition for attentional resources may have confounded measures related to hypercapnia. Future work using VR should emphasize improved fit and a minimal form factor of the head-worn equipment.

Breathing dynamics and CO_2_ retention are known to be sensitive to hormonal effects of menstrual cycle^41^. The present study included 3 female subjects whose menstrual cycle were not determined. Testing sessions were spread across a variable number of days (averaging 8), covering a broad potential range of cycle phases. Female subjects were unlikely to be at the same phase during their time in the study and may have utilized hormone-controlling contraceptives. For these reasons, it is believed unlikely that menstrual hormone effects confounded the data set. Combined oral contraceptive use is common among female astronauts^42,43^, so it is also further unlikely to be relevant to the target contingency scenario.

While the P_I_CO_2_ limit of 30 mmHg is well beyond the predicted maximum a crewmember is likely to see using current space suit hardware^44^, it does not answer the question of where the potential operationally-relevant human limit resides. The question of inflection points is difficult to answer given this limit and the categorical nature of the levels and durations used here. Additional research is warranted that would test higher P_I_CO_2_ levels to model actual tolerance limits.

Summarily, no operationally or clinically relevant physical or cognitive effects were seen during this 1 h simulated contingency EVA walk back scenario during exposure to P_I_CO_2_ levels up to 30 mmHg. The goal of a contingency EVA situation is to return to a habitable volume safely within the time limit of available suit consumables, and here, all subjects were able to complete the task with minimal effects. Conservativism is recommended for contingency EVA Standards with regards to allowable P_I_CO_2_ to account for unknown yet possible impacts of altered gravity, reduced atmospheric pressure, and increased metabolic workload.

## Methods

### Subjects

Subjects were required to be aged 22–55 years old, complete an Air Force Class III-equivalent medical exam, including blood work (complete blood count, comprehensive metabolic panel), a pulmonary function test and chest x-ray to screen out major pulmonary pathology, and exceed a maximum O_2_ uptake (VO_2peak_) cycle ergometry value of >35 mL/kg/min for males; >30 mL/kg/min for females^45^. Exclusion criteria included: a history of smoking, panic attacks, sleep apnea, current use of prescription medications for psychotropic disorders, orthopedic pain, blood pressure or sleep disorders, and pregnancy. The phase of the menstrual cycle was not determined among female subjects. The study was approved by the Institutional Review Board at NASA’s Johnson Space Center (JSC; Study #00000383) and was conducted in accordance with the Declaration of Helsinki. All subjects provided written informed consent and could withdraw at any time without consequence.

### Study design and experimental protocol

The study employed a virtual reality (VR) lunar environment to simulate a contingency EVA “walk back” scenario (i.e., walking back to the habitat for safe haven after experiencing a suit malfunction) during which subjects were exposed to elevated P_I_CO_2_. The scenario deploys a subject two kilometers away from a habitat safe haven, which they must traverse back to in under one hour on a passive treadmill. The P_I_CO_2_ values selected for this study included 0 (dried ambient air control), 5, 10, 15, 20, 25, and 30 mmHg. Each subject underwent one training session for protocol familiarization, followed by seven separate data collections (one for each P_I_CO_2_ level). The P_I_CO_2_ levels were randomized and counterbalanced between subjects, and subjects were blinded to the P_I_CO_2_ level they were exposed to for each session. A minimum of 24 h was required between each test. Subjects engaged in VR-embedded cognitive testing, responded to verbal surveys, and underwent physiological monitoring before, during, and after each data collection session.

All data collections were performed at the NASA Johnson Space Center “Assessments of Physiology and Cognition in Hybrid-reality Environments” (APACHE) facility that combines physical and VR capabilities to create an immersive exploration EVA analog environment^46^. This sea-level laboratory environment was temperature and humidity controlled. Participants walked on a self-powered treadmill (Skillmill Connect, Technogym, Fairfield, NJ) while wearing a Vive Pro Eye VR headset and Vive trackers on ankles (Vive, HTC Corp., Seattle, WA) to translate along a fixed path within the VR Lunar landscape. Vive hand controllers allowed subjects to respond to the embedded cognitive tests. A custom overhead harness (Tuff Tread, Conroe, TX) was worn to support positioning during the treadmill VR traverse, but no gravitational offload was provided. A heads-up display (HUD) within the VR headset was used to provide real-time feedback to the subject related to their mission performance (distance traveled, walking speed, simulated O_2_ depletion rate). A treadmill speed sensor (RUNN Smart, North Pole Engineering, Minneapolis, MN) transmitted speed values to the VR HUD. Participants wore a custom adjustable, 3D-printed Hard Upper Torso (HUT) unit to simulate sensations of spacesuit sizing and chest constriction. Hardware and simulation components are shown in Fig. 4.

The breathing gas mixtures inhaled during the 1-h EVA “walk back” were generated using an air compressor (Kaeser HP28CM, Kaeser Compressors Inc, Fredericksburg, Virginia) and a K-bottle of 100% CO_2_. The desired level of P_I_CO_2_ was accomplished by titrating CO_2_ gas flow into a mixing chamber where it would blend with the compressed, dried, ambient air, before filling two 200-liter Douglas bag (Vacumed 1196-200, Ventura, CA) breathing reservoirs. Subjects breathed from the Douglas bags through a fitted breathing mask (Dräger X-plore 4740, Lübeck, Germany) via 1 3/8-inch (35 mm) diameter tubing. CO_2_ levels were continuously monitored at the exit of the mixing chamber and at the exit of the Douglas bags via Vaisala GMP251 probes (Vaisala, Vantaa, Finland) to ensure that CO_2_ was administered at the appropriate levels. Although the addition of CO_2_ displaces a small amount of oxygen, the hypoxic impact of this dilution as a result of O_2_ dilution was minimal – equivalent to ~340 m altitude in the 30 mmHg (worst case) scenario.

During the familiarization session, subjects were introduced to the environment, equipment, and assessment protocol. During this session, participants completed at least 12 iterations of the VR-embedded Digit Symbol Substitution Tests (DSSTs, described below) to overcome the initial learning curve for cognitive performance assessment. The familiarization session was also used to determine the walking speed bounds within which subjects were expected to maintain their walking speed during data collections. The minimum limit was set to 2.1 km/hr to ensure completion of a 2 km “walk back”, and the maximum speed limit was determined as the walking speed at which the subject’s VO_2_ was 1.3 L/min (about 1600 BTU/hr or 404 kcal/hr), based on their individual VO_2peak_ tests. This limit is 25% higher than the ISS EVA limit to account for additional capabilities of potential future exploration spacesuits.

Prior to each data collection, participants were screened for adequate hydration and sleep, and instructed to avoid large meals in the prior 2 h, greater-than-normal caffeine ingestion in the prior 8 h, any maximal physical activity in the prior 24 h. Before each test session’s “walk back” began, subjects had an opportunity to re-familiarize with the cognitive tests if desired. Subjects then donned the simulated spacesuit HUT. Baseline measurements were then performed, first while subjects were seated and resting for 5 min, followed by donning VR, then taking walking baseline measures on the treadmill for 5 min. Subjects were then introduced to the breathing gas mixture, marking the start of the 1 h “walk back” phase. During this phase, subjects were instructed to maintain cadence within their individual walking speed limits using the VR-displayed speed, perform the cognitive tasks and self-assessments, and terminate the test if they experienced any intolerable symptoms. Cognitive tasks and self-assessments of physical symptoms and performance were administered every 10 min, while physiological data was recorded continuously. Following the “walk back” phase, subjects returned to breathing ambient air in a seated position for recovery up to one hour. Physiological monitoring continued, and symptom assessments were performed every 10 min until the recovery phase ended when symptoms and physiological measures had returned to baseline values or after 1 h, whichever occurred first.

### Measurements

**Fig. 5:**
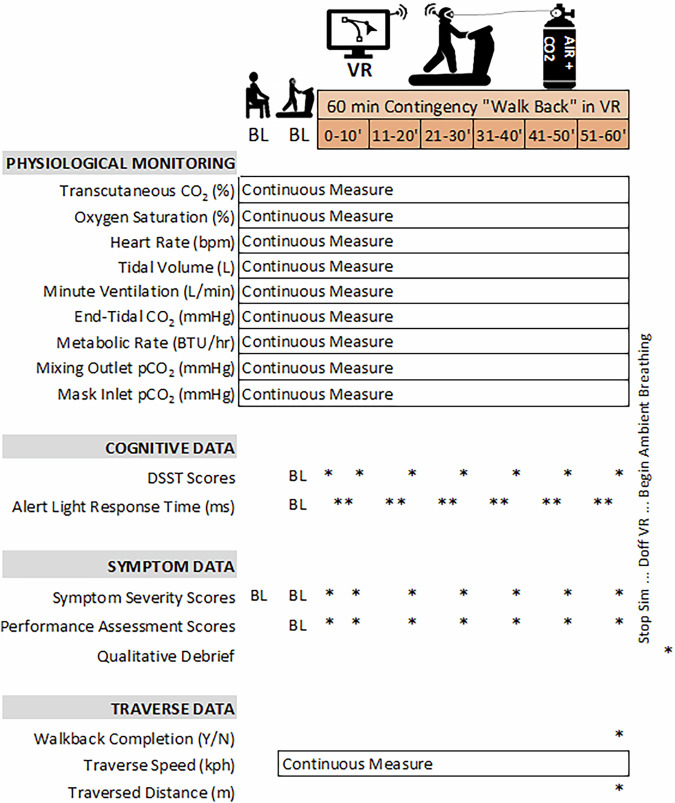
Data collection session design. Data were categorized into Physiological Monitoring, Cognitive Data, Symptom Data, and Traverse Data as shown above. Measurements began with a seated baseline (“BL”) of 5 min followed by a walking baseline data collection of 5 min. Subjects were then instrumented with VR and simulated suit hard upper torso (HUT) hardware and began the 1-h “walk back” scenario with one of 7 randomized levels of P_I_CO_2_.

Each session was comprised of physiological monitoring, measures of cognitive performance, as well as symptom and performance ratings. An overview of a single data collection session is shown in Fig. 5.

Continuous measurements of metabolic rate, heart rate, and pulmonary measures using the COSMED K5 Metabolic Cart (COSMED Srl, Rome, Italy) were collected before, during and after each simulated EVA walkback. Heart rate was measured via the Polar H10 wearable heart rate monitor (Polar Electro, Kempele, Finland) wirelessly integrated with the COSMED K5. Oxygen saturation was measured via the Nellcor PM100-N SpO_2_ Bedside Patient Monitoring System (Covinden LLC, Mansfield, MA) using an adhesive forehead sensor. Transcutaneous CO_2_ (TcCO_2_) was measured via SenTec SDMS (Sentec AG, Therwil, Switzerland) connected to the participant’s earlobe via adhesive ear clip. Walking speed was continuously monitored during the “walk back” period and the total traversal distance was recorded at the end of the period.

For cognitive performance, a combination of validated research and operationally relevant metrics were used to assess performance decrements. VR-embedded cognitive evaluations were provided as a previously established measurement of spatial memory, visual processing, and paired associate learning in terrestrial and spaceflight environments^31,47^. The Digit-Symbol Substitution Test (DSST), shown to be the best predictor of performance on the 6-degrees of freedom spacecraft docking task and modified for the VR environment, occurred at 10-min intervals with 90-second allotments for completion^48^. The DSST was adapted to enable usage in the VR environment. A legend with symbols and numerical digits (1-9) was displayed to the participant’s HUD along with a paired symbol and number below (see Fig. 4H). Responses were given by clicking the Right controller if false and Left controller if true, as quickly as possible, if the individually displayed number-symbol pair matched the one portrayed in the legend. The legend key and number-symbol pair were randomly re-assigned with each administration throughout the 90-second duration. Measurements included proportion of accurate responses (%), number of total responses (n), average reaction time to complete each test pairing (s), and throughput (number of correct responses per minute, n/min). An a priori threshold of a 10% decrease in a given performance outcome was selected to designate a decrement significant enough to potentially impact spaceflight operations. Though arbitrary, this threshold is conservative relative to other studies involving the Cognition battery that tend to demonstrate large effect sizes^29^.

Measurements of reaction time occurred via VR-embedded Alert Light Functional Task^49^. Subjects were presented with a red or green square to left of the nominal HUD (see in Fig. 4G) at two separate, random time intervals in every 10 min block, with these colors requiring a button press of either their right (red light) or left (green light) VR hand controller, and instructed to press the correct button as quickly as possible. The light remained on screen until the correct button was pressed. Alert Light Function Task time was measured based on how quickly (in seconds) the correct button was pressed.

Participants were also verbally queried to assess their own physical symptoms. Symptom intensity was rated on a 0 to 7 Likert scale, with 0 corresponding to “nonexistent symptoms” and 7 corresponding to “intolerable symptoms” for the following symptoms: headache, dizziness, nausea, numbness, tingling, shortness of breath, fatigue, and euphoria. Ratings were recorded during seated baseline, walking baseline, at the onset of the one-hour “walk back”, and every 10 min thereafter until the end of the seated recovery period. Self-assessments of performance were verbally surveyed at 10-min intervals throughout the “walk back” phase, immediately following symptom surveys, including speed performance, endurance performance, and cognitive performance. Speed Performance measured the rating of one’s ability to maintain the self-selected walking speed between the designated bounds, endurance performance measured the perceived stamina relative to normal, and cognitive performance measured the rating of one’s performance on the most recent battery of cognitive tests (DSST and Alert Light Functional Tasks). Participants rated these task performances on a scale of 1–7, with 1 corresponding to “incapable of performing the task” and 7 corresponding to “optimal performance of the task”. The total time and number of incidents in which participants failed to walk at the self-selected pace, as well as the final total distance traversed, were also collected as metrics of physical performance.

### Data analysis

Data were processed via R version 4.3.2 and RStudio (RStudio, PBC, Boston, MA), and analyzed in Stata (StataCorp. 2024. Stata Statistical Software: Release 18.5. College Station, TX: StataCorp LLC). Outliers were removed from data with non-normal distributions if they were more than three standard deviations from the mean of the values. These upper and lower boundaries were determined using MATLAB^®^ (The MathWorks, Inc., Natick, MA, USA).

Outcome measures were analyzed as continuous, normally distributed, mixed effect models, incorporating test condition nested within subject-specific random intercepts to adjust for the repeated measures within subjects. CO_2_ exposure was incorporated as a categorical fixed effect to elucidate the shape of the trend. Effects of Test Order (1–7, incorporated as categorical fixed effect) was also examined to ensure the effectiveness of the CO_2_ level counterbalance. For the cognitive data that was assessed at 10-min intervals during the walk back session, each timepoint was included in the model as a categorical fixed effect. For the Alert Light Function Task data that was assessed randomly during walk back, data were combined into a single category. For the physiological data that was measured continuously over time during testing, data were included in the model as categorical fixed effects for phase of testing (seated baseline, walking baseline, and 1-h CO_2_ intervention). Within-group errors with autoregressive structure was also added to the physiological models to account for the continual measurements over time. Robust standard errors addressed potential non-homogenous variance over exposures and phases. The standardized residuals were visually checked for normality by plotting the quantiles of the residuals against the quantiles of the normal distribution. The Alert Light Functional Task time outcome required a log transformation before modeling to meet the normal assumption. Results were transformed back to the linear scale for reporting. Expected marginal means (LS means) were used to estimate mean trends (including changes from walking baseline), standard errors, and confidence limits. Post hoc contrasts of mean values during the 1-h CO_2_ exposure and same-day, ambient air, walking baseline mean values were also performed to determine the level at which CO_2_ responses were detectably different from ambient air. Mean effect sizes at the 30 mmHg PiCO_2_ exposure are reported with Confidence Intervals in the text of the Results section.

Subject symptom and performance ratings were homogenously nominal (0’s for symptoms, indicating no symptoms; 7’s for performance ratings, indicating optimal performance), and this precluded the mixed modeling approach described above. As such, a descriptive analysis of these measurements was chosen to explore the time course development of any symptoms, as well as within-subject and dose-response trends across CO_2_ levels.

## Data Availability

The data that support the findings of this study are available from the corresponding author upon reasonable request. Moreover, the data can be accessed upon request to the NASA Life Sciences Data Archives.
